# Potential role of gene-environment interactions in ion transport mechanisms in the etiology of renal cell cancer

**DOI:** 10.1038/srep34262

**Published:** 2016-09-30

**Authors:** Ivette A. G. Deckers, Piet A. van den Brandt, Manon van Engeland, Frederik J. van Schooten, Roger W. L. Godschalk, András P. Keszei, Janneke G. F. Hogervorst, Leo J. Schouten

**Affiliations:** 1 Departments of Epidemiology School for Oncology and Developmental Biology (GROW), Maastricht University Medical Centre (MUMC), Maastricht, The Netherlands; 2 Departments of Pathology, School for Oncology and Developmental Biology (GROW), Maastricht University Medical Centre (MUMC), Maastricht, The Netherlands; 3 Department of Toxicology, School for Nutrition, Toxicology and Metabolism (NUTRIM), MUMC, Maastricht, The Netherlands; 4 Department of Medical Informatics, Universitätsklinikum Aachen, Germany; 5 Center for Environmental Sciences, Hasselt University, Diepenbeek, Belgium

## Abstract

We investigated the ion transport mechanism (ITM) in renal cell cancer (RCC) etiology using gene-environment interactions between candidate single nucleotide polymorphisms (SNPs) and associated environmental factors, including dietary intakes of sodium, potassium and fluid, hypertension and diuretic medication. A literature-based selection of 13 SNPs in ten ITM genes were successfully genotyped in toenail DNA of 3,048 subcohort members and 419 RCC cases from the Netherlands Cohort Study. Diet and lifestyle were measured with baseline questionnaires. Cox regression analyses were conducted for main effects and gene-environment interactions. *ADD1*_rs4961 was significantly associated with RCC risk, showing a Hazard Ratio (HR) of 1.24 (95% confidence intervals (CI): 1.01–1.53) for the GT + TT (*versus* GG) genotype. Four of 65 tested gene-environment interactions were statistically significant. Three of these interactions clustered in *SLC9A3*_rs4957061, including the ones with fluid and potassium intake, and diuretic medication. For fluid intake, the RCC risk was significantly lower for high *versus* low intake in participants with the CC genotype (HR(95% CI): 0.47(0.26–0.86)), but not for the CT + TT genotype (*P*-interaction = 0.002). None of the main genetic effects and gene-environment interactions remained significant after adjustment for multiple testing. Data do not support the general hypothesis that the ITM is a disease mechanism in RCC etiology.

Several modifiable risk factors have been confirmed to increase the risk of renal cell cancer (RCC), including obesity, cigarette smoking and hypertension, while increasing evidence suggests that moderate alcohol consumption may be inversely associated with RCC risk^1^. In addition to environmental factors, genetic susceptibility to RCC has been evaluated in numerous candidate-gene association studies, in which several single nucleotide polymorphisms (SNPs) from relevant biologic pathways have been associated with RCC risk^1^ including several genetic variants in genes that are involved in the renin-angiotensin-aldosterone system (RAAS) and hypertension pathways^2^,^3^,^4^. In genome-wide association studies (GWAS), susceptibility loci have been identified near the genes *EPAS1* (*endothelial PAS domain protein 1*; involved in the von Hippel Lindau (VHL)-hypoxia-inducible factor (HIF) oxygen-sensing pathway), *ITPR2* (*inositol 1,4,5-trisphosphate receptor type 2*; associated with waist-hip ratio) and *ZEB2* (*zinc finger E-box binding homeobox 2*; involved in epithelial-mesenchymal transition)^5^,^6^,^7^,^8^,^9^. In a small GWAS among African Americans, an association with RCC risk was reported for a SNP that was located in the *KCNQ2 (potassium voltage-gated channel, KQT-like subfamily, member 2)* gene, but the association was not replicated in the confirmation study^10^. However, in complex diseases, such as RCC, a substantial part of the phenotypic variation may be explained by the modifying effects of genetic variations on lifestyle risk factors (gene-environment interactions)^11^. In addition, gene-environment interactions help to unravel mechanisms underlying carcinogenesis^12^.

Recently, we reported that high sodium intake was associated with a higher risk of RCC^13^ and that this association may differ by genetic variation in genes involved in RAAS^3^, which is a hormonal mechanism that regulates blood pressure and renal sodium homeostasis^14^. Renal sodium homeostasis and the homeostatic balance of other solutes is maintained through renal reabsorption and secretion. This process of ion transport is facilitated by protein carriers or ion-specific channels and is essential, as free diffusion of ions through the renal tubules is limited^14^. Interestingly, these mechanisms have recently been put forward as potential novel mechanism underlying carcinogenesis^15^. Perhaps, the mechanism of ion transport may be another mechanism for which genetic variation may be associated with RCC risk or modify effects of sodium intake in relation to RCC risk.

Ion transport not only involves regulation of sodium homeostasis. Throughout the kidney, renal sodium reabsorption is linked to the reabsorption or secretion of other solutes, such as potassium^14^. Moreover, mechanisms in ion transport are a major focus in the pathogenesis of hypertension, as several forms of antihypertensive therapy intervene on various aspects of ion transport^16^. For example, diuretic medication increases the excretion of fluid and sodium from the kidneys. Therefore, potassium and fluid intake, hypertension and the use of diuretic medication are, in addition to sodium intake, relevant environmental factors in the context of gene-environment interactions in ion transport mechanisms.

In the present study, we used data from the prospective Netherlands Cohort Study (NLCS) on diet and cancer to investigate the association between candidate SNPs in ion transport mechanisms, their interplay with associated environmental factors and RCC risk.

## Results

Genotype and allele frequencies of SNPs in ion transport genes in subcohort members of the NLCS are presented in Table 1. Two SNPs, *SCNN1G*_rs4299163 and *WNK1*_rs10849563, showed a deviation from Hardy-Weinberg Equilibrium (HWE), as tested with Pearson χ^2^ test (*P* < 0.001 and *P* < 0.001, respectively). Despite our priority criteria for multiplex design, i.e. SNPs with minor allele frequency (MAF) ≥20% in Caucasians and SNPs that were not in high linkage disequilibrium (LD) (r^2^ < 0.8), some of the SNPs had a low MAF (*GNB3*_rs4963516; MAF = 0.148) or a high LD (*SCNN1B*_rs239345 and *SCNN1B*_rs11645151; r^2^ = 0.93, D’ = 0.96;) in this study population (Haploview, version 4.2^17^).

At baseline, cases and subcohort members did not substantially differ in dietary intakes (Table 2). However, the proportion of men, cigarette smokers, participants with a diagnosis of hypertension and users of diuretic medication, was higher among cases than among subcohort members. Perceived saltiness, an inverse indicator of salt preference, was higher in cases than in subcohort members. Among subcohort members, there was no association between the three candidate SNPs in two genes that have previously been associated with taste responses in mice, including *SCNN1B* and *SCNN1G*^18^, and perceived saltiness, as tested with the χ^2^ test (range *P*-values = 0.516–0.846).

Out of the 13 investigated candidate SNPs, only *ADD1*_rs4961 was significantly associated with RCC risk (Table 3). Results were similar for the crude and age and sex adjusted model. In the latter model, the HR for the GT + TT (*versus* GG) genotype was 1.24 (95% CI: 1.01–1.53), whereas the HR for each T allele was 1.19 (95% CI: 1.00–1.41). The result was not statistically significant after adjustment for multiple testing.

We tested 65 gene-environment interactions between the candidate SNPs and the exposures under study, including the dietary intakes of sodium, potassium and fluid, hypertension status and the use of diuretic medication, in relation to RCC risk (Table 4). Four gene-environment interactions were significant, but not after adjustment for multiple testing. The correlation between the intakes of sodium, potassium, and fluid, the use of diuretic medication and a history of hypertension was low to moderate among subcohort members (correlations −0.003 to 0.54). Additional adjustment for discretionary salt intake in sensitivity analyses did not change the results.

However, three out of the four observed borderline significant gene-environment interactions included the same SNP, i.e. *SLC9A3*_rs4957061. RCC risk estimates by genotype of this SNP are presented in Table 5. A lower RCC risk was observed for the highest (*versus* the lowest) category of fluid intake in participants with the CC genotype (HR(95% CI): 0.47(0.26–0.86)), whereas no such lower RCC risk was observed for participants with the highest fluid intake and the CT + TT genotype (HR(95% CI): 1.03(0.64–1.65), *P*-interaction = 0.002). This is in line with results from continuous analysis (*P*-interaction = 0.031). For potassium intake, in continuous analyses, the RCC risk for each additional gram of potassium intake per day showed a subtle difference between the CC and CT + TT genotypes (HR(95% CI): 0.77(0.54–1.12) and 1.18(0.94–1.49), respectively; *P*-interaction = 0.028). However, the interaction could not be confirmed in the analyses using tertiles (*P*-interaction = 0.978). Moreover, the interaction between *SLC9A3*_rs4957061 and the use of diuretic medication was statistically significant (*P*-interaction = 0.013). The RCC risk was highest for participants with the CC genotype who used diuretic medication (HR(95% CI): 2.15(1.23–3.75)). High sodium intake (tertiles and increment of 1 g/d) and hypertension were associated with a higher risk of RCC, regardless of the *SLC9A3*_rs4957061 genotype (*P*-interaction = 0.808, 0.827 and 0.399, respectively).

## Discussion

In the present population-based cohort study, we investigated 13 candidate SNPs in ten genes involved in ion transport in relation to RCC risk. Data does not support the general hypothesis that genetic variation in ion transport genes may influence RCC susceptibility, because only one of these SNPs, *ADD1*_rs4961, was significantly associated with RCC risk, but not after adjustment for multiple testing. We additionally investigated potential gene-environment interactions between these genes and exposures that may also be implicated in ion transport in the kidney, including dietary intakes of sodium, potassium and fluid, hypertension and the use of diuretic medication. Although significant gene-environment interactions clustered in one SNP, *SLC9A3*_rs4957061, results were not statistically significant after adjustment for multiple testing, and this does not support the general hypothesis that gene-environment interactions related to ion transport mechanisms are of interest in RCC etiology.

Of the 13 investigated candidate SNPs, only *ADD1*_rs4961 was significantly associated with RCC risk. The *ADD1* gene encodes for α-adducin, a ubiquitously expressed cytoskeleton protein implicated in the formation of actin-spectrin complex, actin polymerization and cell signal transduction^19^,^20^ and has, to our knowledge, not previously been investigated in relation to RCC risk. Lin *et al*. concluded that the G allele of *ADD1*_rs4961 might modulate the decline of renal function in healthy elderly Chinese^21^. In contrast, we observed the highest RCC risk for the GT + TT (*versus* GG) genotype. The *ADD1* gene was selected as candidate gene, because genetic variations in this gene have been associated with hypertension^22^,^23^,^24^. We observed no significant gene-environment interaction between the candidate SNP, *ADD1*_rs4961, and hypertension in relation to RCC risk. In addition, the variant genotype of *ADD1*_rs4961 is believed to induce proximal tubule sodium reabsorption^25^, but we observed no significant interactions between this SNP and sodium intake.

The observed gene-environment interactions were all clustered in one SNP, i.e. *SLC9A3*_rs4957061. The *SLC9A3* gene encodes the Na^+^/H^+^ exchanger 3 (NHE3), which is the major sodium transporter on the apical membrane of the renal proximal tubule cells^26^. This is of particular importance for RCC, as the majority of RCC tumors are thought to arise from the proximal renal tubule^27^. Animal studies, as well as studies in hypertensive patients suggest that this gene is important in the control of blood pressure through its effect on sodium transport^28^,^29^,^30^. It is however remarkable that the observed gene-environment interactions for *SLC9A3*_rs4957061 did not include hypertension or sodium intake, but potassium intake, fluid intake and use of diuretic medication. In Cox regression analyses, RCC risk estimates for fluid intake were similar in both continuous and categorical analyses; the highest intake was associated with a lower RCC risk for participants with the *SLC9A3*_rs4957061 reference genotype and not for participants with the variant genotype. Previously, we reported that there was no overall association between fluid intake and RCC risk present in this study population, after 17.3 years of follow-up^13^.

Some of the candidate SNPs investigated in the present study are SNPs in *SCNN1B* and *SCNN1G.* These genes may be involved in the salty taste transduction pathway in the KEGG pathway database^31^ and have previously been associated with salt taste responses in mice^18^. Therefore, these genes may be of particular importance in the association between sodium intake and RCC risk. We hypothesised that SNP-related interindividual variation in the *SCNN1B* and *SCNN1G* genes may determine one’s perceived saltiness and ultimately the actual sodium intake. We could not confirm our hypothesis, because none of the SNPs in *SCNN1B* and *SCNN1G* were associated with perceived saltiness in the subcohort.

Several methodological considerations should be noted as to interpret the results of the present study. Given the prospective study design and the high ascertainment of RCC cases, selection bias and information bias are unlikely in the NLCS. The long follow-up provided sufficient number of cases for the current analyses, but may also have introduced some random misclassification regarding dietary intakes, which were calculated from a single measurement at baseline. In addition, it is possible that the proportions of participants with hypertension and diuretic medication use may slightly be underestimated using this single measurement. Both might have resulted in attenuation of the investigated gene-environment interactions. Moreover, different action mechanisms of various diuretics (e.g. potassium sparing) may have influenced the results. Information on the exact type of diuretic medication was not available. Furthermore, RCC is a heterogeneous disease, with respect to histology and (epi)genetic aberrations. It is conceivable that the observed associations were attenuated, because we did not take these subtypes into account. Finally, the results of two SNPs in particular, i.e. *SCNN1G*_rs4299163 and *WNK1*_rs10849563, should be interpreted with care, as they were not in HWE in our subcohort. However, there is no reason to assume that this error is different for cases and subcohort members or for different categories of the investigated exposures. Therefore, this potential genotyping error would rather have led to missing a true interaction (if any) than detecting an interaction^32^.

For the present study, we used a candidate SNP approach and selected SNPs based on prior knowledge from current literature. Our initial SNP selection rendered too many potential SNPs of interest for the limited space available on the multiplex design. The 13 SNPs in ten ion transport genes that were successfully genotyped cover only a fraction of the large and complex network of genetic and environmental factors in which the investigated SNPs are included. As a result, true effects might still be diluted or masked by effects of those not included in the present study. In addition, the null results in the present study do not exclude the possibility that other SNPs in these genes or SNPs in other genes related to ion transport may influence RCC susceptibility. New studies in other populations are required to investigate this hypothesis.

In conclusion, results from the present study show some first indications of involvement of *ADD1*_rs4961 and *SLC9A3*_rs4957061 in RCC susceptibility. However, results do not support the general hypothesis that the mechanism of ion transport is a major underlying disease mechanism in RCC etiology, since the selected SNPs in ion transport genes and gene-environment interactions between these SNPs and related exposures were mostly not associated with RCC risk.

## Methods

### Study design, study population and follow-up

The Netherlands Cohort Study on diet and cancer (NLCS) is a prospective cohort study initiated in 1986 when 58,279 men and 62,573 women between the ages of 55–69 years were included^33^,^34^,^35^. The case-cohort design was used for efficiency in questionnaire processing and follow-up. Cases were derived from the entire cohort, whereas a subcohort of 5,000 subjects was randomly sampled at baseline to estimate person years at risk^36^. Subcohort members were regularly followed up for vital status information. All cohort members were followed up for cancer occurrence through computerized record linkage with the Netherlands cancer registry and the Dutch pathology registry (PALGA)^37^. The coverage of cohort members by the Netherlands cancer registry and PALGA to establish cancer follow-up is estimated to be over 96%^38^. Cases and subcohort members with prevalent cancer (excluding skin cancer) at baseline were excluded. After 20.3 years of follow-up, 608 microscopically confirmed RCC cases were identified (International Classification of Diseases for Oncology 3 (ICD-O-3): C64).

The NLCS has been approved by the institutional review boards of the TNO Quality of Life Research Institute (Zeist, the Netherlands) and Maastricht University (Maastricht, the Netherlands). All methods were carried out in accordance with the approved guidelines. All cohort members consented to participate in the study and the use of the biological samples by completing and returning the self-administered questionnaire and a bag with toenail clippings.

### Toenail DNA for genotyping

Approximately 90,000 participants provided toenail clippings at baseline. DNA was isolated from these toenails as described previously^39^. To increase our case sample, for 67 RCC cases without toenail clippings, DNA was isolated from formalin-fixed paraffin-embedded (FFPE) normal kidney tissues, as described by van Houwelingen *et al*.^40^ and added to the sample collection of toenail DNA. Toenail DNA has shown to be a valid source of DNA for the genotyping of a limited set of SNPs when using the SEQUENOM^®^ MassARRAY^®^ platform using the iPLEX TM assay (Sequenom, Hamburg, Germany)^41^.

### SNP selection and multiplex design

Genes were of interest if they were involved in ion transport mechanisms in the kidney and have been associated with the regulation of blood pressure or hypertension. The following candidate genes were included: *adducin1* (*ADD1*), *ATPase Na**+**/K**+** transporting alpha and beta 1 polypeptide* (*ATP1A1* and *ATP1B1*), *guanine nucleotide binding protein (G protein), beta polypeptide 3* (*GNB3*), *regulator of G-protein signaling 5* (*RGS5*), *sodium channel non-voltage-gated 1 beta and gamma subunit* (*SCNN1B* and *SCNN1G*), *solute carrier family 9, subfamily A* (*NHE3*, *cation proton antiporter 3*) *member 3* (*SLC9A3*), *serine threonine kinase 39* (*STK39*) and *WNK lysine deficient protein kinase 1* (*WNK1*). For these genes, SNPs were selected through extensive literature search considering the number of previously reported associations with i) the risk of RCC, ii) the risk of hyper- or hypotension iii) systolic or diastolic blood pressure, iv) enzymes or proteins in the renin-angiotensin-aldosterone system and v) salt handling, ion transport or salt sensitivity. This search strategy rendered over 100 potential SNPs of interest. A maximum of 40 SNPs could be included in the multiplex design. We gave priority to SNPs with more than one previously reported association, a MAF ≥ 20% in Caucasians (because of power considerations) and to SNPs that were not in high LD with each other (r^2^ < 0.8). SNPs with the highest priority were first allocated to the assay. Nevertheless, due to potential sequence incompatibilities among primers, not all high priority SNPs could be included in the design. For example, we included *WNK1_*rs10849563 (A > G) as a proxy for our high priority SNP *WNK1_*rs765250 (T > C, r^2^ = 1.0 and D’ = 1.0), which has been associated with essential hypertension and blood pressure, but could not be combined with other SNPs on the multiplex. Subsequently, SNPs with less priority were used to fill the multiplex. Our final multiplex design included 31 SNPs. This manuscript focuses on gene-environment interactions with respect to the ion transport mechanism, including 13 SNPs from 10 genes. Gene-environment interactions with SNPs from the RAAS pathway have been reported in a separate manuscript^3^.

### SNP genotyping

**Figure 1:**
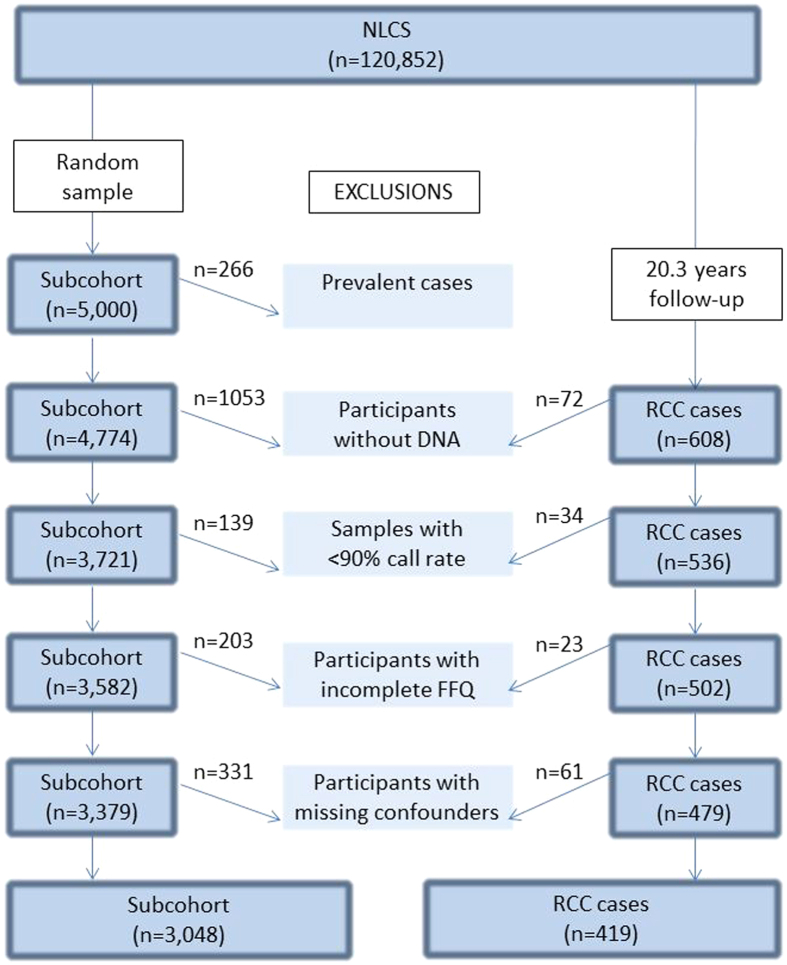
Flow chart of members of the Netherlands cohort study on diet and cancer (NLCS) for analyses after 20.3 years of follow-up, 1986–2006. Used abbreviations: FFQ = Food-Frequency Questionnaire. RCC = Renal cell cancer.

For one SNP, *ADD1*_rs17833172, the genotyping completely failed for unknown reasons. The remaining 30 SNPs were successfully genotyped: 96% of the samples had a call rate of at least 90% and the genotype concordance between duplicates and between toenail samples and FFPE healthy tissues samples was respectively 99.6% and 99.1% on average (n = 23). For 4,066 samples corresponding to 3,582 subcohort members and 502 RCC cases, complete genotyping data were available for further analyses (see Fig. 1). All SNP call rates were at least 99.9%, except for *SCNN1G*_rs4299163, *SLC9A3*_rs4957061 and *WNK1_*rs10849563, which had a SNP call rate of 95.4%, 94.0% and 98.7%, respectively (Table 1).

### Baseline questionnaire

All participants returned a mailed, self-administered, baseline questionnaire on diet and other risk factors for cancer. This baseline questionnaire included a 150-item, semi-quantitative food frequency questionnaire (FFQ), which was used for the assessment of dietary habits. Participants with an incomplete or inconsistent FFQ were excluded, leaving 3,379 subcohort members and 479 cases eligible for further analyses^33^. The FFQ ranked individuals adequately according to dietary intakes when compared to a 9-day dietary records^33^, and reflected nutrient intakes for at least 5 years^34^. Nutrient intakes were calculated using the Dutch food composition table 1986–87^42^. The daily sodium, potassium and fluid intakes were defined as intakes through both foods and beverages per day, including amounts naturally present in foods and beverages plus amounts added during food processing by food manufacturers. Sodium and potassium intake were adjusted for total energy intake by using the residual-mean method^43^ and modelled as sex-specific tertile distributions. Fluid intake was modelled as categorical variable using the categories low (≤1.75 L/d), moderate (1.75–2.25 L/d) and high (>2.25 L/d) intake.

In addition to the FFQ, the baseline questionnaire included specific questions on salt added during home-preparation and before consumption (i.e. discretionary salt intake), which could not be measured using the 150 food items^13^,^44^. Furthermore, the baseline questionnaire included two questions on the saltiness of restaurant food and soups from package or can (ranging from “not salty enough” to “much too salty”), which were used to assess salt preference, as this may influence total salt (sodium) intake. Finally, participants were asked to report whether they had ever been diagnosed with ‘high blood pressure’ by a physician, whether they used any medication for more than six months and, if so, for which medical condition they used what kind of medication. Diuretic medication was defined according to the Anatomical Therapeutic Chemical (ATC) classification of the WHO Collaborative Centre for Drug Statistical Methodology^45^,^46^.

### Statistical analyses

All analyses were conducted using Stata version 12 (Stata Corp., College Station, TX). HRs and 95% CI for the independent main effects of each SNP on RCC risk were evaluated in a crude model and an age and sex adjusted model using Cox proportional hazards analyses adjusted for the case-cohort design^47^. Genotypes were analysed in a dominant model to maintain optimal power and in an allelic model. In subcohort members, we evaluated whether SNPs in genes that have previously been associated with taste responses in mice, including *SCNN1B* and *SCNN1G*^18^, were associated with perceived saltiness at baseline using the χ^2^ test.

Gene-environment interactions including the candidate SNPs and the intakes of sodium, potassium and fluid, hypertension and the use of diuretic medication were tested using the Wald χ^2^ test by including the interaction term into the model. Risk estimates were only presented for the significant gene-environment interactions. Interaction analyses were performed in a multivariable-adjusted model, including *a priori* selected potential confounders: age at baseline (years), sex (male/female), total energy intake (kcal/day), body mass index (BMI, kg/m^2^), cigarette smoking (status (non-current/current), intensity (cigarettes/day) and duration (years)), alcohol consumption (g ethanol/day), hypertension (no/yes) and use of diuretic medication (no/yes). Analyses including sodium intake were additionally adjusted for discretionary salt intake (g/d) in sensitivity analyses. Participants with missing information on confounders were excluded, leaving 3,084 subcohort members and 419 cases available for multivariable analysis.

For all the models including covariates, the proportional hazards assumption was tested using the scaled Schoenfeld residuals. A violation was apparent for age, which was therefore modelled as time dependent covariate. All tests were two-sided and a *P*-value < 0.05 was considered statistically significant. To adjust for multiple testing, we applied the false discovery rate (FDR) approach of Benjamini-Hochberg^48^. The FDR was set at 20%, which is common in candidate gene studies^49^. FDR-adjusted P-values were separately calculated for the main genetic effects and for the interactions.

## Additional Information

**How to cite this article**: Deckers, I. A. G. *et al*. Potential role of gene-environment interactions in ion transport mechanisms in the etiology of renal cell cancer. *Sci. Rep.* **6**, 34262; doi: 10.1038/srep34262 (2016).

## Figures and Tables

**Table 1:** Characteristics of SNPs in ion transport genes in the subcohort members of the Netherlands cohort study, 1986–2006.

SNPs in salt transporter genes	Chr. location	Ref. alleles	Minor allele	MAF	Genotypes						HWE^b^
					11^a^		12^a^		22^a^		
					n	%	n	%	n	%	
*ADD1*_rs4961	4p16.3	G/T	T	0.212	2,232	62.33	1,182	33.01	167	4.66	*0.52*
*ATP1A1*_rs850602	1p21	C/G	G	0.236	2,089	58.35	1,290	36.03	201	5.61	*0.93*
*ATP1B1*_rs2901029	1q24	A/G	G	0.432	1,176	32.83	1,716	47.91	690	19.26	*0.15*
*GNB3*_rs1129649	12p13	C/T	C	0.351	1,536	42.87	1,582	44.15	465	12.98	*0.07*
*GNB3*_rs4963516	12p13	A/C	C	0.148	2,607	72.76	894	24.95	82	2.29	*0.60*
*RGS5*_rs2815287	1q23.1	A/C	C	0.377	1,371	38.27	1,4718	47.96	493	13.76	*0.23*
*RGS5*_rs2999967	1q23.1	C/T	C	0.461	1,057	29.51	1,750	48.86	775	21.64	*0.31*
*SCNN1B*_rs11645151	16p12.2-p12.1	C/T	C	0.251	1,993	55.70	1,371	38.32	214	5.98	*0.31*
*SCNN1B*_rs239345	16p12.2-p12.1	A/T	A	0.252	1,994	55.67	1,368	38.19	220	6.14	*0.51*
*SCNN1G*_rs4299163	16p12	C/G	C	0.207	2,193	64.01	1,050	30.65	183	5.34	*<0.001*
*SLC9A3*_rs4957061	5p15.3	C/T	T	0.470	942	28.13	1,664	49.69	743	22.19	*0.89*
*STK39*_rs6749447	2q24.3	G/T	G	0.268	1,928	53.88	1,379	38.54	271	7.57	*0.27*
*WNK1*_rs10849563	12p13.3	A/G	G	0.306	1,748	49.45	1,413	39.97	374	10.58	*<0.001*

^a^Genotypes 11, 12 and 22 refer to the homozygote common, heterozygote and homozygote rare genotype, respectively.

^b^*P*-value for Hardy-Weinberg equilibrium.

**Table 2:** Baseline characteristics of subcohort members and renal cell cancer cases in the Netherlands Cohort Study, 1986–2006.

Baseline characteristics (mean (sd))	Subcohort members	RCC Cases
Total (N)	3379	480
Dietary intakes (in g/d)		
Sodium^a^	2.3 (0.6)	2.5 (0.7)
Discretionary salt^b^,^c^	2.8 (2.7)	2.8 (3.0)
Perceived saltiness (%)		
Not salty enough	1.9	2.0
Good	41.9	36.7
A little too salty	41.6	41.2
Much too salty	14.7	20.2
Potassium^a^	3.5 (0.6)	3.6 (0.6)
Fluid (L/d)	2.1 (0.5)	2.1 (0.5)
Potential confounders		
Age (y)	61.3 (4.2)	61.0 (4.0)
Male sex (%)	49.8	65.2
Total energy intake (kcal/d)	1925 (506.2)	1994.0 (515.9)
Alcohol consumption (g ethanol/d)^d^	13.7 (15.0)	15.7 (16.1)
BMI (kg/m^2^)^c^	25.0 (3.1)	25.5 (3.0)
Cigarette smoking status: current (%)	27.4	31.9
Duration in current smokers (y)	38.7 (8.7)	39.6 (8.5)
Intensity in current smokers (cigarettes/d)	14.7 (8.7)	15.9 (8.7)
Hypertension (%)	26.2	31.9
Use of diuretic medication (%)	10.8	13.1

Abbreviations: BMI, body mass index; RCC, renal cell cancer; sd, standard deviation.

^a^Intakes are energy-adjusted.

^b^Salt intake refers to sodium-chloride intake (sodium-chloride = sodium ∗ 2.54).

^c^N does not correspond with the overall N, due to missing values.

^d^In consumers only.

**Table 3:** Associations between SNPs in ion transport genes and renal cell cancer risk in the Netherlands Cohort Study, 1986–2006.

SNPs in ion transport genes	Person years^a^	RCC Cases	Crude model			Age and sex adjusted model			
			HR	95% CI	p-value	HR	95% CI	p-value	FDR-adjusted p-value
*ADD1*_rs4961									
GG	32,542	240	1.00	(ref)		1.00	(ref)		
GT + TT	19,590	178	1.23	(1.00–1.52)	0.049	1.24	(1.01–1.53)	0.045	0.533
T^b^	52,133	418	1.17	(0.99–1.38)	0.072	1.19	(1.00–1.41)	0.050	0.484
*ATP1A1_*rs850602									
CC	30,051	239	1.00	(ref)		1.00	(ref)		
CG + GG	22,064	179	1.02	(0.83–1.25)	0.853	1.03	(0.84–1.27)	0.789	0.946
G^b^	52,115	418	1.06	(0.89–1.26)	0.503	1.07	(0.90–1.27)	0.444	0.831
*ATP1B1_*rs2901029									
AA	17,296	135	1.00	(ref)		1.00	(ref)		
AG + GG	34,847	283	1.04	(0.84–1.30)	0.713	1.07	(0.85–1.33)	0.577	0.938
G^b^	52,143	418	0.97	(0.84–1.12)	0.696	0.99	(0.86–1.14)	0.904	0.904
*GNB3_*rs1129649									
TT	22,427	192	1.00	(ref)		1.00	(ref)		
CT + CC	29,729	227	0.89	(0.73–1.10)	0.278	0.90	(0.73–1.10)	0.302	0.654
C^b^	52,156	419	0.94	(0.81–1.10)	0.454	0.96	(0.82–1.12)	0.576	0.831
*GNB3_*rs4963516									
AA	38,191	293	1.00	(ref)		1.00	(ref)		
AC + CC	13,965	126	1.18	(0.94–1.47)	0.158	1.15	(0.92–1.45)	0.216	0.654
C^b^	52,156	419	1.18	(0.96–1.43)	0.110	1.16	(0.95–1.42)	0.149	0.484
*RGS51_*rs2815287									
AA	20,255	151	1.00	(ref)		1.00	(ref)		
AC + CC	31,887	267	1.12	(0.91–1.39)	0.283	1.13	(0.91–1.40)	0.257	0.654
C^b^	52,142	418	1.12	(0.96–1.30)	0.153	1.12	(0.96–1.30)	0.142	0.484
*RGS5_*rs2999967									
TT	15,541	111	1.00	(ref)		1.00	(ref)		
CT + CC	36,594	308	1.18	(0.94–1.49)	0.163	1.23	(0.97–1.56)	0.082	0.533
C^b^	52,135	419	1.10	(0.95–1.27)	0.188	1.13	(0.98–1.31)	0.089	0.484
*SCNN1B_*rs11645151									
TT	28,984	235	1.00	(ref)		1.00	(ref)		
CT + TT	23,084	184	0.98	(0.80–1.21)	0.876	0.99	(0.80–1.22)	0.914	0.946
C^b^	52,069	419	1.04	(0.87–1.23)	0.677	1.04	(0.87–1.24)	0.676	0.831
*SCNN1B_*rs239345									
TT	29,081	233	1.00	(ref)		1.00	(ref)		
AT + AA	23,075	186	1.01	(0.82–1.24)	0.949	1.01	(0.82–1.24)	0.946	0.946
A^b^	52,156	419	1.04	(0.88–1.23)	0.644	1.04	(0.87–1.23)	0.684	0.831
*SCNN1G*_rs4299163^c^									
GG	31,805	248	1.00	(ref)		1.00	(ref)		
GC + CC	17,990	146	1.04	(0.84–1.29)	0.721	1.05	(0.84–1.30)	0.691	0.946
C^b^	49,795	394	0.99	(0.83–1.18)	0.911	0.99	(0.83–1.17)	0.896	0.904
*SLC9A3_*rs4957061									
CC	13,797	109	1.00	(ref)		1.00	(ref)		
CT + TT	35,129	297	1.07	(0.85–1.35)	0.576	1.09	(0.86–1.38)	0.501	0.930
T^b^	48,926	406	0.96	(0.84–1.11)	0.613	0.97	(0.84–1.12)	0.703	0.831
*STK39_*rs6749447									
TT	28,255	240	1.00	(ref)		1.00	(ref)		
GT + GG	23,819	179	0.89	(0.72–1.09)	0.248	0.88	(0.71–1.08)	0.224	0.654
G^b^	52,075	419	0.90	(0.76–1.06)	0.212	0.90	(0.76–1.06)	0.200	0.520
*WNK1_*rs10849563^c^									
AA	25,065	200	1.00	(ref)		1.00	(ref)		
AG + GG	26,333	213	1.01	(0.82–1.25)	0.903	1.01	(0.82–1.24)	0.937	0.946
G^b^	51,398	413	0.97	(0.83–1.12)	0.662	0.96	(0.83–1.12)	0.639	0.831

Abbreviations: RCC, renal cell cancer; SNPs, single nucleotide polymorphisms.

^a^In the subcohort.

^b^Trend for genotype based on the allelic model.

^c^HWE assumption has not been met for this SNP (See Table 1).

**Table 4:** Summary of *P* values for interaction between dietary intakes, hypertension and use of diuretic medication and SNPs in ion transport genes for renal cell cancer risk in the Netherlands Cohort Study, 1986–2006.

SNPs in ion transport genes^c^	*P* for interaction^a,b^				
	Sodium intake (g/d)^d^	Potassium intake (g/d)^d^	Fluid intake (L/d)	Use of diuretic medication	Doctors’ diagnosis of hypertension
*ADD1*_rs4961	*0.101*	*0.279*	*0.346*	*0.875*	*0.990*
*ATP1A1_*rs850602	*0.336*	*0.684*	*0.847*	*0.535*	*0.091*
*ATP1B1_*rs2901029	*0.854*	*0.363*	*0.710*	*0.605*	*0.187*
*GNB3_*rs1129649	*0.355*	*0.942*	*0.706*	*0.276*	***0.026***^e^
*GNB3_*rs4963516	*0.518*	*0.529*	*0.895*	*0.633*	*0.552*
*RGS5_*rs2815287	*0.353*	*0.061*	*0.701*	*0.863*	*0.692*
*RGS5_*rs2999967	*0.742*	*0.723*	*0.356*	*0.285*	*0.356*
*SCNN1B_*rs11645151	*0.481*	*0.275*	*0.797*	*0.326*	*0.498*
*SCNN1B_*rs239345	*0.507*	*0.268*	*0.647*	*0.699*	*0.376*
*SCNN1G*_rs4299163	*0.233*	*0.069*	*0.860*	*0.059*	*0.109*
*SLC9A3_*rs4957061	*0.827*	***0.028***^e^	***0.031***^e^	***0.013***^e^	*0.399*
*STK39_*rs6749447	*0.279*	*0.751*	*0.498*	*0.147*	*0.338*
*WNK1_*rs10849563	*0.496*	*0.727*	*0.881*	*0.290*	*0.068*

^a^Using Wald χ^2^ test for interaction.

^b^Interaction adjusted for age at baseline (years), sex (male/female), total energy intake (kcal per day), smoking status (non-current/current), smoking intensity (cigarettes per day), smoking duration (years), BMI (Kg per m^2^), alcohol intake (grams of ethanol per day), hypertension (yes/no) and use of diuretic medication (yes/no).

^c^SNPs are analyzed in a dominant model.

^d^Intakes are energy-adjusted by using the residual mean method.

^e^FDR-adjusted p-value: 0.504.

**Table 5:** Interactions between dietary intakes, hypertension and use of diuretic medication and *SLC9A3*_rs4957061 for renal cell cancer risk in the Netherlands Cohort Study, 1986–2006.

	Person years	*SLC9A3_*rs4957061							
		CC			CT + TT			*P*-interaction^b^	FDR-adjusted p-value
		Cases	HR^a^	95% CI	Cases	HR^a^	95% CI		
Sodium intake^c^,^d^									
T1	16,402	30	1.00	(ref)	85	1.19	(0.77–1.87)		
T2	16,621	34	1.13	(0.66–1.91)	92	1.25	(0.80–1.95)		
T3	15,903	45	1.69	(1.02–2.79)	120	1.67	(1.09–2.56)	*0.808*	
Increment of 1 g/d	48,926	109	1.33	(0.91–1.93)	297	1.20	(0.99–1.45)	*0.827*	*0.923*
Potassium intake^c^,^d^									
T1	15,969	36	1.00		87	1.07	(0.70–1.63)		
T2	16,642	33	1.03		104	1.11	(0.73–1.68)		
T3	16,316	40	1.06	(0.65–1.73)	106	1.20	(0.79–1.81)	*0.978*	
Increment of 1 g/d	48,926	109	0.77	(0.54–1.12)	297	1.18	(0.94–1.49)	*0.028*	*0.504*
Fluid intake									
Low	11,281	28	1.00	(ref)	59	0.82	(0.50–1.34)		
Moderate	21,686	58	1.01	(0.61–1.67)	120	0.81	(0.51–1.28)		
High	15,959	23	0.47	(0.26–0.86)	118	1.03	(0.64–1.65)	*0.002*	
Increment of 1 L/d	48,926	109	0.60	(0.36–1.02)	297	1.10	(0.83–1.46)	*0.031*	*0.504*
Hypertension									
No	36,292	68	1.00	(ref)	204	1.18	(0.88–1.59)		
Yes	12,635	41	1.57	(1.01–2.42)	93	1.49	(1.03–2.15)	*0.399*	*0.810*
Use of diuretic medication									
No	43,944	85	1.00	(ref)	267	1.25	(0.96–1.62)		
yes	4,982	24	2.15	(1.23–3.75)	30	1.16	(0.71–1.87)	*0.013*	*0.504*

^a^HRs are adjusted for age at baseline (years), sex (male/female), total energy intake (kcal per day), smoking status (non-current/current), smoking intensity (cigarettes per day), smoking duration (years), BMI (Kg per m^2^), alcohol intake (grams of ethanol per day), hypertension (yes/no) and use of diuretic medication (yes/no).

^b^Using Wald χ^2^ test for interaction.

^c^Tertile distribution is sex-specific and based on subcohort members.

^d^Intakes are energy-adjusted by using the residual mean method.
